# Differences between behavioral time budget and welfare indicators in two different slow-growing broiler genotypes kept in the free-range system

**DOI:** 10.1007/s11259-025-10814-9

**Published:** 2025-06-28

**Authors:** Arda Sözcü, Aydın İpek, Stefan Gunnarsson

**Affiliations:** 1 Faculty of Agriculture, Animal Science Department, Bursa Uludağ University, Bursa, 16059 Türkiye; 2 Animal Environment and Health Department, Swedish University of Agricultural Sciences (SLU), Uppsala, 53223 Sweden

**Keywords:** Feather pecking, Foot pad dermatitis, Free-range, Tonic immobility, Walking ability, Slow growing broiler

## Abstract

The consumer interest for meat from slow growing broilers in free-range system has increased recently. Therefore, the need for knowledge about behaviour and welfare of birds in these systems has increased. The aim of this study was to compare the differences between behavioral time budget, tonic immobility and clinical welfare indicators in two slow growing broiler genotypes (Hubbard ISA Red JA-57 and Sasso XL44 × SA51A) kept in a free-range system. In total, 480 one-day old chicks were reared, and the birds were regularly scored for behavioral time budget and multiple welfare indicators. The eating and drinking tended to decrease in Sasso birds, whereas they showed an increment in Hubbard birds with increasing of age (*P* < 0.01). Hubbard birds had the highest percentage of explorative pecking (7.65%) of the total time budget compared to the Sasso birds (4.33% at day 63, *P* < 0.01). Comb pecking wounds, skin injuries and gait scores were affected by both genotype and age (*P* < 0.01). The duration of tonic immobility was found to be longer, as well as the number of tonic immobility inductions was higher in Sasso birds compared to the Hubbard (26.49 vs. 19.68 s; 1.54 and 1.24, respectively *P* < 0.01). These findings indicate that birds of the Hubbard genotype may be more prone to comb pecking and skin injuries, but they showed less fearful and higher walking ability, compared to Sasso birds.

## Introduction

Continuous genetic selection of broilers has resulted in a higher body weight, a more effective feed conversion and increased breast muscles, and this selection has been primarily focused on economic traits to reduce cost of production (Akyüz et al. 2022, 2024; Korver 2023). This selection for rapid growth has made the modern broilers as the fastest growing species among farm animals (Ghayas et al. 2021). However, such a rapid growth also seems to be associated with problems in health, behavior, and welfare in broilers.

In modern and intensive production systems, broilers are reared at high stocking density in confined houses, and the birds reach the slaughter weight within approximately 40 days (Korver 2023). However, this rapid growth may result in impaired welfare regarding with behavioral problems, including locomotion problems with poor walking ability (Hartcher and Lum 2020). However, the stocking density of broilers in conventional systems and high body weight negatively affect the walking ability (Shynkaruk et al. 2023). One of the most important health and welfare concerns is leg disorders which could be assessed with walking ability of birds (Kwon et al. 2024). It has been highlighted that the production system heavily effects skeletal development and bone disorders by Çapar Akyüz and Onbaşılar (2020). Around 30% of all broilers reared in intensive production systems have some form of leg disorders (EU 2016). These negative issues have caused needs for finding solutions for improving the welfare of broiler chickens. In recent years, the consumer demands for chickens reared in free-range and organic systems have increased, as these systems could decrease the stressful conditions found in conventional production systems, and thereby can increase the comfort and improve the welfare status and behavioral patterns of commercial birds (Wang et al. 2009).

To meet minimum husbandry standards: a slow-growing broiler genotype must be used and kept for a longer rearing period, as well as feeds with low content of fat and high content of cereals, the birds are kept at lower stocking rates and should have access to a pasture area (Amato and Castellini 2022). Free-range systems are enriched as pasture covered by natural or artificial vegetation for the birds with free access (Chen et al. 2013). Accessing to pasture areas make possible to exhibit the natural behaviors of birds, for example, scratching, foraging, dust bathing, sunbathing, perching and activity, stimulate more physical activity and utilize the natural daylight and sunshine (Ipek and Sozcu 2017). Behaviors, e.g., pecking, scratching, walking, and resting, reflect the emotional status of birds, and are used as welfare indicators (Welfare Quality Consortium 2009). Depending on the increment of physical activity and growth pattern of slow-growing birds, growth and strength of bones takes shape well and leg disorders could be prevented largely (Mikulski et al. 2011).

There are many drop effecting factors for welfare status, behavioral patterns and subsequently performance, that are also depending on the rearing system, genotype, sex, feed ration, physical activity, management, and environmental conditions (Gordon and Charles 2002; Varol Avcılar et al. 2018; Erbay Elibol et al. 2021; Eser et al. 2022; Tekin Demir et al. 2024; Gündoğar et al. 2024). To improve the welfare status of free-ranging birds, genotype should be correctly chosen regarding with their ability to use the pasture and foraging (Australian Egg Corporation 2012). The relationship between range use and behavior is still unclear (Campbell et al. 2018, 2019; Ferreira et al. 2019, 2020a). Ferreira et al. (2019, 2020b) highlighted that variation in ranging behavior could affect chicken behavior, thusly chickens ranged less inhibited their behavior when compared to the chickens ranged more.

This could be also useful to exhibit the natural behaviors such as foraging and sunbathing etc., and physical activity (Riber et al. 2018). Therefore, investigating and comparing of welfare and behavioral patterns of birds are crucial for choosing of appropriate genotypes that could be recommended for producers and to develop a better management standard in free-range system. Therefore, studies focused on welfare, behavior and pasture usage of different genotypes could be useful to improve health and welfare status, and by that improve environmental sustainability that can also support successful production (Marchewka et al. 2020).

The present research was carried out as a part of the FreeBirds project that aim is to develop better husbandry practices in organic poultry production by encouraging the birds to be more at outdoor and achieve a better agreement with the intentions of the organic concept. Besides, Bonnefous et al. (2023) emphasized that genetics could be effective for behavioral consistency during a bird’s life. This study compared the differences between behavioral time budget, and clinical welfare indicators in chickens from two slow growing broiler genotypes (Hubbard ISA Red JA-57 and Sasso XL44 × SA51A) kept in the free-range system. In the study, two slow growing genotypes that have similar growth rate, slaughter age were selected to make an objective comparison to observe the differences in respect of genetic variability.

## Materials and methods

### Animals and housing

This study was performed in Research Farm of Department of Animal Science, Faculty of Agriculture, Bursa Uludağ University (Bursa, Türkiye). In the study, a total of 480 one-day old chicks (equal ratio for male and female) of two slow growing genetic line (Hubbard ISA Red JA-57 and Sasso XL44 × SA51A, at 42 weeks of age) were kept in a free-range system. The chicks were weighed at the beginning of the experiment by using a balance at ± 0.1 g precision, and thereafter they were randomly allocated into six experimental pens (*n* = 3 pens/genotype, 80 birds/pen) with a floor area of 3 × 7 m^2^. The space allowance was provided as 0.26 m^2^/bird (changed between 12 and 14 kg per m^2^) in the pens. The experimental period was 63 days, and carried out during autumn months.

The pens had an outdoor pasture area that was regulated according to the minimum standards in EU Directive 1999/74/EC (European Union Directive 1999/74/EC). The indoor floor in the pens was covered with wood shavings as litter material. Lighting program was applied according to optimum standards given by EU Directive 1999/74/EC (European Union Directive 1999/74/EC). Circular plastic feeders, plastic bell drinkers and wooden perches (18 cm perch length/bird) were provided indoor solely. In each of pen, indoor and outdoor areas were separated by a solid wall with a small pop-hole (60 × 60 cm) to provide free access to pasture. The pasture area (350 m^2^/pen) was limited by wire fences to keep out predators and each pen had an artificial shade cloth shelter with green color and with a size of 5 × 7 m. The stocking density was 4.4 m^2^ per bird in the pasture area.

### Data collection

To determine the behavioral time budget of the broilers, the back of four randomly sampled birds from each pen was marked with green paint. Marking of birds was regularly refreshed during the experimental period. Behavioral observations were performed on four times during the growing period at 28, 42, 56 and 63 days of age.

The direct observations were performed on the observation days at 9.00–11.00 h and 15.00–17.00 h, respectively, by the same observer and in both the indoor and outdoor areas. The observer sat or stood in a position outside the pens with a clear view of the entire pen under observation. Each pen of the six pens was scanned separately at 10 min intervals, thus giving 12 records per pen. The numbers of birds in a pen performing each of the behaviors eating, drinking, preening, feather pecking, walking–standing, explorative pecking, and resting-lying were sequentially recorded as a series of instantaneous scans. The definitions of behaviors recorded were modified from Zhao et al. (2014) (Table 1). Furthermore, during the behavioral observations, the average value of maximum distance of the birds from the house was determined as the distance reached by the birds. The total spent time at outdoor was calculated as the percentage of birds at pasture area with respect to the total number of broilers in each pen.

**Table 1:** The ethogram used in the present study, including the definitions of the different behaviors modified by Zhao et al. (2014)

Behaviors	Definition
Eating	Bird has its beak in contact with feed repeatedly/once
Drinking	Bird has its beak in contact with drinkers or raises its head when swallowing water
Preening	Bird has its beak in contact with its own plumage, performing movements of pecking, combing, rotating, or nibbling once or repeatedly
Feather pecking	Bird pecks the feathers of conspecifics
Walking-standing	Bird moves with a normal or quick speed or stands in a stationary position
Explorative pecking	Bird pecks other object in the house, except feathers
Resting	Bird lies on its abdomen or sits with its legs under the body

Each pen of the six pens was scanned separately at 10 min intervals

In a tonic immobility (TI) test, a total of 36 birds per genotype (12 from each pen) were randomly selected and tested individually for response at 28, 42, 56 and 63 days of age. The hens were taken from their home pen and carried to another room. All tests were performed by the same researcher, and individual signings with wing-tagged method was used to prevent repeated testing of the same bird. To measure the duration of TI, the birds were caught randomly and carried to a separate room. A few seconds after the broiler was caught, TI was induced according to Ghareeb et al. (2014). The experimenter put one hand on the hen’s chest and another over its head, letting the head dangle down, and restrained the hens for five seconds while they were on their backs in a metal cradle. The lone experimenter then took their hands off their hands and went aside, looking down. After five minutes of immobilization or when the bird straightened itself after at least ten seconds, whichever came first, the test was over. If the hen righted itself in less than 10 s, the restraint was repeated up to five times, and the duration of tonic immobility was noted. A maximum of three inductions was applied and the maximum test duration was set to 600 s. The total duration of TI was recorded as the time until the bird stood in an upright position.

A range of clinical welfare indicators was assessed for all bird at 28, 42, 56 and 63 days of age using the Welfare Quality^®^ protocol (Welfare Quality Consortium 2009) (Table 2). All birds in each pen was scored for comb pecking wounds, plumage condition, enteritis, skin injuries, footpad dermatitis, hock burn, missing toes, and nails. Furthermore, each broiler was individually evaluated for walking ability by using a gait score ranging from 0 to 5 (Table 3; (Welfare Quality Consortium 2009). For the scoring, the evaluator sat on the pen floor at eye level viewing the back of the broiler’s legs while it walked for 15 s. After completing the scoring, each of the score were calculated as a percentage value for each genotype.

**Table 2:** Welfare indicators used for welfare assessment in the present study, including the definitions of the scores

Item	Score		
	0	1	2
Comb pecking wounds	No wounds	One or two wounds	Three or more wounds
Plumage conditions, body*	Intact feathers	Moderate wear (< 5 cm)	At least 1featherless area > 5 cm in diameter
Enteritis	Absent	Present	-
Skin injuries	< 3 pecks or scratches	Lesions < 2 cm or > 3 pecks or scratches	At least 1 lesion > 2 cm
Footpad dermatitis (FPD)	No lesion	Mild: Small lesion ≤ 0.2 cm	Severe: Larger lesion > 0.2 cm
Hock burn	No lesion	Small lesions, necrosis, or proliferation of epithelium, but no or moderate swelling	Visible inflammation or swelling
Missing toes/nail	Absent	Present	-

*The scores for each body part (breast, back, and tail) were combined to give a total plumage score

Source: Welfare Quality R^®^ (2009)

**Table 3:** Scoring of walking ability of broilers

Score	Mean
0	Normal, agile, and well-balanced
1	Slight abnormality and uneven walking but difficult to define
2	Uneven walking with shortened steps, failure in balance, taking support from the wings, definite and identifiable abnormality
3	Obvious abnormality, affects ability to move, not standing for more than 15 s, after walking lying down
4	Severe abnormality, unwilling to walk, using wings as crutches, only takes a few steps
5	Incapable of walking

Source: Welfare Quality R^®^ (2009)

At 63 days of age, blood samples were collected from randomly selected 6 broilers for each genotype. To determine the leukocytes, a smear of blood was placed on a glass slide for each bird. The slides were stained with May-Grünwald and Giemsa stains, and then counted with a light microscope (model BX41TF, Olympus Corporation, Tokyo, Japan) at ×100 magnification (Gross and Siegel 1983). For each slide, one hundred leukocytes (heterophiles, eosinophils, basophils, lymphocytes and monocytes) were counted, and then the H/L ratio was calculated by dividing the number of heterophils by that of lymphocytes (Salamano et al. 2010).

### Statistical analysis

All data were analyzed with the mixed model procedure in the statistical analysis software SAS (version 9.4 2012 Cary NC USA). Data was analysed at pen level on logscale, and normality of the data was assessed based on model residuals. Behavioural data was aggregated and expressed as percentage of broilers performing a certain behavioural category compared to total number of birds observed for eating, drinking, preening, feather pecking, walking-standing, explorative pecking and resting. Data regarding welfare indicators (comb pecking wounds, plumage condition, enteritis, skin injuries, FPD, hock burn, missing toes/nail, gait score) and tonic immobility were recorded as a coherent data for each broiler separately. The comparative analysis of the genotypes and the broiler age were performed with a univariate analysis of variance. Non-parametric statistical test were performed with the Kruskal-Wallis test. All data were analysed using linear mixed models consisting of fixed effects of genotype and broiler age, and the interactions between genotype × broiler age. Pen was included as a random effect. Analyses of percentage data were conducted after arcsine square root transformation of the data. The blood parameters were subjected to the t-test procedure in SAS (version 9.4 2013 Cary NC USA). Significant differences between means were compared using the Tukey test and were considered statistically different at *P* < 0.05.

## Results

The percentages of the behaviors performed by the two genotypes (Hubbard and Sasso) at different ages (28, 42, 56 and 63 days of age) are shown in Table 4. With increasing of age, the eating and drinking showed a decreasing tendency in Sasso genotype, whereas it showed a decline (at 42 and 56 days) and then an increment in Hubbard genotype at 63 days of age (*P* < 0.01). At 63 days of age, a higher percentage of eating and drinking were observed in Hubbard broilers than Sasso ones (17.73 vs. 15.52%, and 4.02 vs. 2.71%, respectively, *P* < 0.001). The preening and feather pecking increase significantly in both of genotypes with increasing age (*P* < 0.05). The highest percentage of explorative pecking was observed in Hubbard genotype (7.65%), whereas the lowest percentage was found in Sasso genotype at 63 days of age (4.33%, *P* < 0.001). The walking-standing behavior showed a significant variation in both Hubbard and Sasso birds while the birds aged (*P* < 0.01). A higher percentage of walking and standing was observed in Sasso birds compared to the Hubbard birds (21.14% vs. 17.73%, *P* < 0.05). Furthermore, resting-lying behavior was significantly lower at 63 days of age in both of genotype compared to the young ages (46.77% and 48.66%, respectively, *P* < 0.05). Birds of the Hubbard genotype walked significantly longer distances from the house than the Sasso birds (10.95 vs. 6.65 m, *P* < 0.01).

**Table 4:** Behavioral observations for two slow growing broiler genotypes in the free-range system

Main factors	Eating	Preening	Drinking	Explorative pecking	Walking-Standing	Feather pecking	Resting- Lying	Maximum distance from the house (m)	Total spent time at outdoor (%)
	(%)								
Genotype									
Hubbard	16.97	2.00 ^b^	3.51	6.27 ^a^	14.18 ^b^	2.50	54.57	10.95 ^b^	31.58 ^a^
Sasso	16.99	2.24 ^a^	3.36	5.95 ^b^	14.84 ^a^	2.48	54.14	6.65 ^a^	7.74 ^b^
SEM	0.16	0.10	0.12	0.13	0.26	0.09	0.22	1.10	1.56
Age (days)									
28	17.77 ^a^	1.42 ^c^	3.38	6.39	14.65 ^b^	1.82 ^c^	54.58 ^c^	4.55 ^b^	15.33 ^b^
42	16.99 ^b^	1.85 ^b^	3.50	6.01	12.62 ^c^	2.12 ^bc^	56.92 ^b^	8.62 ^ab^	17.33 ^ab^
56	16.63 ^b^	2.16 ^b^	3.49	6.04	11.34 ^d^	2.22 ^b^	58.21 ^a^	11.85 ^a^	22.67 ^a^
63	16.54 ^b^	3.05 ^a^	3.37	5.99	19.44 ^a^	3.82 ^a^	47.72 ^d^	10.20 ^a^	23.32 ^a^
SEM	0.22	0.15	0.17	0.19	0.37	0.12	0.32	1.54	2.21
Genotype × Age									
Hubbard × 28	17.45 ^abc^	1.33 ^c^	3.23 ^bc^	6.20 ^bcd^	14.53 ^c^	2.03 ^bc^	55.23 ^cd^	6.80	22.33 ^b^
Sasso × 28	18.09 ^a^	1.51 ^c^	3.53 ^ab^	6.58 ^b^	14.76 ^c^	1.60 ^c^	53.93 ^d^	2.30	8.33 ^c^
Hubbard × 42	16.52 ^cde^	1.82 ^bc^	3.73 ^abc^	5.64 ^cd^	12.98 ^cd^	2.17 ^bc^	57.51 ^ab^	10.50	26.33 ^b^
Sasso × 42	17.46 ^abc^	1.89 ^bc^	3.63 ^ab^	6.38 ^bcd^	12.25 ^d^	2.06 ^bc^	56.33 ^bc^	6.73	8.33 ^c^
Hubbard × 56	16.19 ^de^	2.32 ^b^	3.41 ^abc^	5.58 ^d^	11.47 ^d^	2.24 ^b^	58.78 ^a^	14.73	37.33 ^a^
Sasso × 56	16.88 ^bcd^	2.01 ^bc^	3.57 ^ab^	6.49 ^bc^	11.20 ^d^	2.21 ^b^	57.64 ^ab^	8.97	8.00 ^c^
Hubbard × 63	17.73 ^ab^	2.52 ^b^	4.02 ^a^	7.65 ^a^	17.73 ^b^	3.57 ^a^	46.77 ^f^	11.80	40.33 ^a^
Sasso × 63	15.52 ^e^	3.57 ^a^	2.71 ^c^	4.33 ^e^	21.14 ^a^	4.06 ^a^	48.66 ^e^	8.60	6.30 ^c^
SEM	0.31	0.21	0.19	0.26	0.53	0.17	0.45	2.18	3.12
*p*-values									
Genotype	0.928	0.029	0.170	0.026	0.024	0.806	0.072	0.001	< 0.001
Age	< 0.001	< 0.001	0.700	0.142	< 0.001	< 0.001	< 0.001	0.002	0.005
Genotype × Age	< 0.001	0.002	< 0.001	< 0.001	< 0.001	0.014	< 0.001	0.854	0.001

^a–f^ Means in the column with different letters differ significantly (*p* < 0.05)

As the birds became older, the maximum distance from the house showed an increment from 4.55 m at 28 days of age to 10.20 m at 63 days of age (*P* < 0.02). It changed from 4.55 m at 28 days of age to 10.20 m at 63 days of age. A significant genotype × age interaction was observed for the total spent time at outdoor (*P* < 0.01). Hubbard genotype broilers spent more time outdoor, whereas Sasso birds spent less time, with increasing of age. At 63 days of age, the total spent time at outdoor was found as 40.33% and 6.30% in Hubbard and Sasso birds respectively.

The results from the TI test are shown in Table 5. No significant interactions (genotype × age) were observed for number of TI inductions, as well as TI duration. The duration of TI was found to be longer and the number of inductions were higher in Sasso birds compared to the Hubbard birds (26.49 vs. 19.68 s; 1.54 and 1.24, respectively *P* < 0.01). On the other hand, as with increasing age, the duration of TI showed an increment, and it was changed from 12.68 s at 28 days of age to 35.79 s at 63 days of age (*P* < 0.01).

**Table 5:** Results of the tonic immobility (TI) tests for two slow growing broiler genotypes in the free-range system

Main factors	TI duration (s)	Number of TI Inductions
Genotypes		
Hubbard	19.68 ^b^	1.24 ^b^
Sasso	26.49 ^a^	1.54 ^a^
SEM	1.78	0.08
Age (days)		
28	12.68 ^c^	1.30
42	19.38 ^bc^	1.25
56	24.45 ^b^	1.45
63	35.79 ^a^	1.57
SEM	2.51	0.12
*p*-values		
Genotype	0.002	0.003
Age	< 0.001	0.078
Genotype × Age	0.691	0.882

^a–c^ Means in the column with different letters differ significantly (*p* < 0.05)

TI duration refers to length of tonic immobility episodes; number of TI inductions refers to number of tonic immobility episodes

A significant effect of genotype on comb pecking wounds, skin injuries, and gait score was observed (*P* < 0.05 Table 6). A higher severity of comb pecking wounds and skin injuries was observed in Hubbard birds, whereas the mean of gait score was found to be higher in Sasso birds. On the other hand, the increasing age affected all of welfare indicators (*P* < 0.01). As expected, the severity of each welfare indicator was worse at 63 days of age, when compared to the 28 days of age.

**Table 6:** Welfare indicators of two slow growing broiler genotypes in the free-range system

Main factors	Comb pecking wounds	Plumage condition	Enteritis	Skin injuries	FPD	Hock burn	Missing toes/nail	Gait score
Genotypes								
Hubbard	0.94 ^a^	1.03	0.39	0.59 ^a^	0.85	0.85	0.31	0.88 ^b^
Sasso	0.66 ^b^	1.03	0.38	0.29 ^b^	1.13	1.13	0.27	1.04 ^a^
SEM	0.11	0.08	0.04	0.05	0.15	0.17	0.05	0.04
Age (days)								
28	0.38 ^c^	0.13 ^c^	0.17 ^b^	0.14 ^c^	0.30 ^b^	0.30 ^b^	0.02 ^c^	0.00 ^d^
42	0.65 ^bc^	0.80 ^b^	0.30 ^b^	0.38 ^b^	0.57 ^b^	0.57 ^b^	0.27 ^b^	0.67 ^c^
56	0.95 ^ab^	1.50 ^a^	0.52 ^a^	0.57 ^ab^	1.50 ^a^	1.50 ^a^	0.32 ^b^	1.40 ^b^
63	1.22 ^a^	1.67 ^a^	0.56 ^a^	0.67 ^a^	1.60 ^a^	1.60 ^a^	0.55 ^a^	1.77 ^a^
SEM	0.16	0.11	0.06	0.06	0.21	0.21	0.06	0.06
*p*-values								
Genotype	0.021	0.985	0.757	< 0.01	0.069	0.064	0.375	0.001
Age	< 0.01	< 0.01	< 0.01	< 0.01	< 0.01	< 0.01	< 0.01	< 0.01
Genotype × Age	0.836	0.851	0.380	0.299	0.187	0.895	0.763	0.133

^a–d^ Means in the column with different letters differ significantly (*p* < 0.05)

The differential cell count results and H/L ratio as a stress indicator at 63 days of age was given in Table 7. No significant differences were observed for lymphocytes, monocytes, basophils and eosinophils of Hubbard and Sasso genotypes (*P* > 0.05), whereas a higher percentage of heterophiles and H/L ratio were observed in Sasso birds compared to the Hubbard birds (respectively 40.1% vs. 37.7%, 1.69 vs.1.51, *P* < 0.05).

**Table 7:** The differential cell count results and H/L ratio of two slow growing broiler genotypes in the free-range system

Genotypes	Differential cell count (%)					H/L
	Heterophiles	Lymphocytes	Monocytes	Basophils	Eosinophils	
Hubbard	37.7	25.0	10.7	13.3	13.3	1.51^b^
Sasso	40.1	23.7	11.0	12.7	12.2	1.69^a^
SEM	0.57	1.08	0.82	0.78	0.92	0.08
*p*-values	0.013	0.205	0.643	0.643	0.251	0.045

For counting analysis, randomly sampled 6 birds from each genotype were used at 63 days of age

## Discussion

The current results clearly showed that the genotype and age affected their behavioural time budget and range usage preference in the free-range system. The most frequent behaviours were eating, walking-standing and resting-lying at all ages in both of genotypes. There is a relationship between eating and drinking (Savory et al. 1978). In this study, a higher percentage of eating behaviour was observed at the early ages, and it showed a decline while the birds aged in both of genotypes. Under normal conditions, it is expected an increment in feed consumption due to increasing body weight. However, in this study, eating behaviour shows decrease over time, which may depend on that the birds were eating more during when they were in the ranging as they spent more time at outdoor area with increasing age. It showed a significant increase in ranging behaviour from 15.33% at 28 days of age to 23.32% at 63 days of age in both strains.

Preening is one of the most important comfort behaviors in birds and helps keeping their plumage in good condition (Sandilands et al. 2004). The current results showed that Hubbard and Sasso birds showed preening behavior with a percentage of 2.52% and 3.57%, respectively at 9 weeks of age. The reason for this could be that the plumage is less developed in young broiler chickens, or that other behaviors are more important than preening for birds of young age. In this study there were significant effects of both genotype and the age on preening behavior.

It is well known that due to mutual effects between feather pecking and stressful conditions, the occurrence and severity of feather pecking could be accepted an indicator for reduced welfare of birds, especially in laying hens (Huber Eicher and Sebo 2001). However, the proportions of feathered and feather-free body parts are important parameters to evaluate the energy and nutrient requirements of growing broilers (Wecke et al. 2017). The current results clearly showed that the tendency of genotypes for explorative pecking and feather pecking changed by age. The feather pecking showed an increment in both of Hubbard and Sasso broilers with aging of birds. These findings clearly emphasized the importance of foraging area and some enrichment of this area to be attractive for birds to minimize the pecking behavior which could be related aggression and stressfulness.

The behaviors of walking-standing and resting-lying were the most common observed behaviors. Interestingly, the walking-standing showed an increment, whereas the resting-lying behavior decreased by increasing of age in both of genotype. It is contradictory with previous studies indicated that slow-growing broilers became inactivity and less active with age (Göransson et al. 2021). This could be related with lower daily body weight gain of slow-growing broilers raised in the free-range system and mild severity of lameness (Ferrante et al. 2009). Observed higher walking-standing behavior by age could be related with minor gait impairments and a higher interest against outdoor area and foraging in the study. Thus, these findings could be supported with increment of maximum distance from the house and the total time spent at outdoor with age in Hubbard and Sasso.

Tonic immobility is a common measure of stress and fearfulness, because of TI duration refers to the alertness or fearfulness of the birds (Hata et al. 2018). It has been reported that a shorter duration of TI shows that birds are more alert and respond more quickly to possible dangers (Ghayas et al. 2021). According to this hypothesis, Hubbard broilers were more alert compared to the Sasso birds with a longer duration of TI indicating higher levels of fearfulness. Furthermore, the TI duration showed increment by increasing of age due to increment in body weight. This could be also related with increasing of movement related to increment in walking and standing behavior at outdoor, maximum distance from the house and the total spent time at outdoor.

Our results demonstrated that Hubbard broilers had a higher prevalence and more severe comb pecking wounds and skin injuries. Previous studies indicated that feather pecking, and cannibalistic pecking could cause skin injuries (Cloutier et al. 2000; Lambton et al. 2015). On the other hand, Sasso broilers had moderate walking defects that were determined by gait score. These results showed that Hubbard genotype was more susceptible for pecking problems, whereas Sasso genotype had a higher susceptibility for walking problems in free-range system. Furthermore, the mean score of all the measured welfare indicators showed increment by age in both genotypes.

In the free range systems, the birds are potentially exposed to various stressors related with a huge variation of environmental conditions and free access to outdoor (Bergmann et al. 2017). The heterophiles and lymphocytes have crucial importance for innate and adaptive immunity (Minias 2019; Mosca et al. 2019). Stressful conditions cause an increment heterophiles and a decline in lymphocytes, therefore H/L is accepted an indicator for resistance to diseases and ability to cope with stressful conditions (Thiam et al. 2022). Stefanetti et al. (2023) clearly demonstrated that there has been a huge difference for H/L ratio between broiler genotypes (fast growing vs. slow growing) and also production system (conventional system vs. alternative system). It has been reported that a higher value of H/L (1.69) observed in Ross broilers kept in free range system could be caused a higher stress level and accepted an indicator for their lower ability to cope with environmental stimulants, compared to other local genotypes called as Bionda Piemontese, Robusta Maculata, Bionda Piemontese x Sasso, Robusta Maculata x Sasso. On the other hand, local genotypes or slow growing broiler genotypes are potentially adaptable or free-range system with a lower H/L ratio and mortality (Fiorilla et al. 2023). In this study, Hubbard broilers had a lower H/L ratio compared to Sasso broilers, showing that Hubbard broilers could have less stress level under free range conditions.

In conclusion, this present study found some significant differences in behavioral time budget and welfare status of the slow-growing broilers (Hubbard and Sasso) and compare the suitability of these genotypes in respect to various indicators for free-range system. In general, Hubbard birds seems to be more advantageous in free-range systems for foraging and range use at outdoor area but displayed more aggressive behaviors e.g. pecking. However, Sasso birds were more fearful, and this may affect the range use and foraging behavior negatively. Genetic differences found should be considered in future studies on how to minimize the negative effects of aggression and fearfulness of birds and improve the range use under commercial conditions in large broiler flocks.

## Data Availability

No datasets were generated or analysed during the current study.
